# Obesity Is Associated with Early Onset of Gastrointestinal Cancers in California

**DOI:** 10.1155/2018/7014073

**Published:** 2018-09-19

**Authors:** Yen-Yi Juo, Melinda A. Maggard Gibbons, Erik Dutson, Anne Y. Lin, Jane Yanagawa, O. Joe Hines, Guido Eibl, Yijun Chen

**Affiliations:** ^1^Center for Advanced Surgical and Interventional Technology (CASIT), University of California, 757 Westwood Plaza, Suite B-792, Los Angeles, CA 90095, USA; ^2^Department of Surgery, University of California, Box 956904, 72-251 Center for Health Sciences, Los Angeles, CA 90095, USA; ^3^Department of Surgery, George Washington University, 2150 Pennsylvania Ave, NW, Suite 6B, Washington, DC 20037, USA

## Abstract

**Background:**

Although it is well known that obesity is a risk factor for gastrointestinal (GI) cancer, it is not well established if obesity can cause earlier GI cancer onset.

**Methods:**

A cross-sectional study examining the linked 2004–2008 California Cancer Registry Patient Discharge Database was performed to evaluate the association between obesity and onset age among four gastrointestinal cancers, including esophageal, gastric, pancreatic, and colorectal cancers. Regression models were constructed to adjust for other carcinogenic factors.

**Results:**

The diagnosis of obesity (BMI > 30) was associated with a reduction in diagnosis age across all four cancer types: 3.25 ± 0.53 years for gastric cancer, 4.56 ± 0.18 years for colorectal cancer, 4.73 ± 0.73 years for esophageal cancer, and 5.35 ± 0.72 for pancreatic cancer. The diagnosis of morbid obesity (BMI > 40) was associated with a more pronounced reduction in the age of diagnosis: 5.48 ± 0.96 years for gastric cancer, 7.75 ± 0.30 years for colorectal cancer, 7.67 ± 1.26 years for esophageal cancer, and 8.19 ± 1.25 years for pancreatic cancer. Both morbid obesity and obesity remained strongly associated with earlier cancer diagnosis for all four cancer types even after adjusting for other available cancer risk factors.

**Conclusions:**

The diagnosis of obesity, especially morbid obesity, was associated with a significantly earlier gastrointestinal cancer onset in California. Further research with prospective cohort data may be required to establish the causal relationship between obesity and cancer onset age.

## 1. Introduction

The obesity epidemic has been growing at a steady rate in the United States. Between 1960 and 2006, the number of the overweight population has doubled, while the obese population increased five-fold [1]. Within the obesity epidemic, a subcohort of morbidly obese (BMI > 40) or super obese (BMI > 50) patients has been growing at an alarming rate. Between 1986 and 2000, the prevalence of subjects with BMI over 30 roughly doubled, while the prevalence of subjects with a BMI over 40 quadrupled, and the prevalence of BMI over 50 quintupled [2]. Due to the numerous chronic conditions associated with obesity, about 26,000 excess deaths per year have been attributed to obesity [3].

In addition to contributing to heart disease, stroke, and diabetes, obesity is a major contributor to the nation's cancer toll. It has quickly overtaken tobacco as the leading preventable cause of cancer. Each year, as many as 84,000 cancer diagnoses are attributed to obesity and being overweight and obesity are implicated in 15% to 20% of total cancer-related mortality [4]. Obesity has been found to be associated with increased incidence of a variety of cancers, including postmenopausal breast [5, 6], prostate [6], colorectal [7, 8], esophageal adenocarcinomas [9], gastric [9], pancreas [10], liver [11], and melanomas [12]. Cancer is predicted to overtake heart disease as the leading cause of death in the United States by 2030 [13]. Recently, the American Society of Clinical Oncology published its first ever position statement on obesity and cancer to increase education and awareness and help oncology providers to treat obesity in cancer patients [4].

Up to now, no studies have examined the link between obesity and early cancer onset due to the lack of data with sufficient longitudinal follow-up. Each cancer is usually associated with a specific age distribution. For example, gastric cancers are commonly found in patients above 55 years old [14], and colorectal and pancreatic cancers are usually diagnosed above 65 years old [15, 16], while esophageal cancer is most commonly diagnosed between 45 and 70 years old [17]. Recently, researchers have observed a gradual increase in incidence of early-onset colorectal cancers [18] concomitant with the rise in obesity prevalence, raising the question whether the observed earlier onset of cancers may be attributable to obesity. Recognition of early cancer risk factors are very important because patients with exposure to those risk factors may need earlier and more frequent cancer screening. As a cancer risk factor, we hypothesize that obesity is associated with a younger age of onset for GI cancers and this association correlates with the severity of obesity. To test this hypothesis, we investigated the relationship between obesity and four of the most common GI cancers including esophageal, gastric, colon, and pancreatic cancer diagnosis age using two population-based databases in the state of California between 2002 and 2008.

## 2. Material and Methods

A retrospective review of all incident cases of the four most common GI cancers (esophageal, gastric, pancreatic, and colorectal) among inpatients in California between 2002 and 2008 was performed. The California Cancer Registry (CCR) is a statewide cancer surveillance system that captures all new cancer diagnoses by statute. Cancer onset age and demographic information were obtained from CCR, which was then linked to the California's Patient Discharge Database (PDD) in order to obtain information regarding obesity status and coexistent carcinogenic risk factors. PDD was assembled by the Office of Statewide Health Planning and Development (OSHPD) and documents discharge information from all acute care hospitals licensed by the state of California. The linkage was performed by programmers at the CCR using probabilistic data-matching techniques based on Social Security Number (SSN), date of birth, and gender. Deidentified data files were then provided to the research team after the linkage.

Inclusion criteria were any patient diagnosed with esophageal, gastric, pancreatic, and colorectal cancer between 2002 and 2008 and admitted to a hospital in the state of California. Patients with one of the four cancers of interest were identified from the CCR by *International Classification of Diseases for Oncology* (ICD-O) code. Patients with carcinoma in situ (stage 0) had prior cancer history on the same organ or had missing value with regard to age or diagnosis age were excluded from the analyses.

### 2.1. Variables of Interest

The primary exposure of interest was obesity. Patients within the PDD database with obesity were identified using the *International Classification of Diseases, Ninth Revision, Clinical Modification* (ICD-9-CM) code 278.00, signifying obesity, defined as a body mass index of greater than 30 kg/m^2^. Morbid obesity was identified using ICD-9-CM code 278.01, signifying a body mass index of greater than 40.

The primary outcome of interest was the patient's age at the time of primary cancer diagnosis, which was derived from deducting the birth date from diagnosis date within CCR. Secondary outcome was early onset cancer, defined as primary cancer diagnosis at an age of less than 50 years.

Additional covariates were those demographic factors and medical comorbidities considered contributory to carcinogenesis for each respective cancer in the literature. Demographic factors included gender and race/ethnicity. Medical comorbidities included tobacco dependence, alcoholism, and inflammatory bowel disease. Information regarding insurance availability and access to cancer screening for individual patients was not available in the database.

### 2.2. Statistical Analysis

Descriptive analyses were performed for all available data and tabulated. Bivariate tables were constructed to compare baseline demographics and comorbidities by obesity status for the full sample and within each cancer type. The age at cancer diagnosis was summarized as mean and standard deviation, under the premise that the underlying distribution assimilates normal distribution. Categorical variables were examined for independence using chi-squared tests and continuous variables were compared using Student's *t*-tests. In order to quantify the independent association between obesity status and cancer onset age, multivariate regression models were constructed to adjust for confounding effects of available known carcinogenic factors. All available cancer risk factors from the database were included in the regression models with no differential variable selection algorithm. As PDD data were clustered within sampled hospitals, standard errors were estimated with cluster-robust Huber-White estimators in our regression models. Data analyses were executed using Stata MP Version 13.0 (StataCorp; College Station, TX). All reported *p* values were two-sided with values less than 0.05 considered as significant. Due to the deidentified retrospective nature of the data, this study was considered from review by the UCLA institutional review board.

## 3. Results

A total of 173,585 inpatients were diagnosed with one of the four GI cancers between 2002 and 2008 in the state of California. Among these, 3,611 patients were excluded due to previous history of cancer on the same organ and 167 patients excluded due to unknown birth or diagnosis age, leaving 169,807 patients for analysis. Of these, 158,758 (93.5%) were classified as nonobese, 8,312 (4.9%) as obese, and 2,737 (1.6%) as morbidly obese. The characteristics of the study participants are reported in Table 1, stratified by cancer type. Among the four GI cancers, colorectal cancer patients had the highest rates of obesity (5.7%) and morbid obesity (1.9%), while esophageal cancer patients had the lowest rates of obesity (3.1%) and morbid obesity (0.9%). Due to the small sample sizes observed for morbidly obese patients, we have elected to perform subsequent analyses combining both genders as one group instead of performing separate analyses for males and females. Onset age distribution appeared to approximate normal distribution for all four cancer types; therefore, *t*-tests were employed for subsequent analyses.

**Figure 1:**
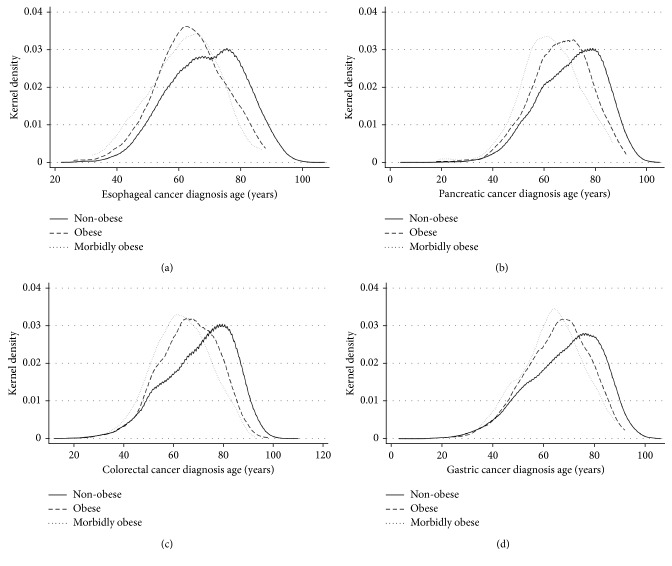
Kernel density plot of diagnosis age distribution for four gastrointestinal cancers. Kernel density plots of cancer diagnosis age was produced to estimate the relative age distribution of nonobese, obese, and morbidly obese patients who had esophageal (a), pancreatic (b), colorectal (c), and gastric cancer (d). Due to the disparate sample size between BMI groups, kernel density, a nonparametric estimation of the age distribution, was employed to create visual representations of the age distributions to allow comparison.

A consistent association between onset age and obesity was found for all four GI cancers. Obese patients (BMI > 30) had a younger onset age than nonobese patients, and morbidly obese patients (BMI > 40) had an even younger onset age (see Table 2 & Figure 1). Patients who were diagnosed with GI cancer before the age of 50 were referred to have early onset GI cancer. The percentage of early onset cancer was also highest among morbidly obese patients, followed by obese and nonobese patients (Table 2). Obesity and morbid obesity caused the most significant increase of early cancer in esophageal cancer: a 56% increase in obese patients and 166% increase in morbidly obese patients (Table 2). The strength of the association between obesity and age of cancer diagnosis appeared to be correlated with the severity of obesity.

The American Joint Commission on Cancer stage (AJCC, 6th edition), tumor histology, race, gender, and other risks factors of cancer information were tabulated by cancer type (Supplementary Tables 1 to 4). As expected, obese and morbidly obese patients had a higher prevalence of diabetes. However, there were no consistent correlations between other cancer risk factors and diagnosis of obesity. Despite an earlier cancer diagnosis, obese and morbidly obese patients did not have lower AJCC cancer stage at the time of diagnosis.

Regression models were constructed for each cancer type in order to examine the independent association between obesity and onset age for the four GI cancers after adjusting for other known etiologic factors. The findings were compatible with the above-described results from the bivariate analyses. When diagnosis age was examined as a continuous variable using linear regression models, the diagnosis of obesity was found to be independently associated with a lower diagnosis age across all four cancer types, with a reduction in age of diagnosis ranging from 3.25 years in gastric cancer to 5.35 years in pancreatic cancer. The diagnosis of morbid obesity was associated with a reduction in the age of diagnosis ranging from 5.48 years in gastric cancer to 8.19 years in pancreatic cancer (see Table 3). Other common cancer risk factors such as alcoholism, male gender, and smoking were all associated with earlier age of diagnosis for all four GI cancers. Ulcerative colitis and Crohn's disease significantly decreased the onset age for colon cancer: 8.45 ± 0.56 years and 7.45 ± 0.79 years earlier, respectively.

## 4. Discussion

The present study represents one of the first population database-based studies demonstrating decreased mean GI cancer onset age among obese patients in comparison with nonobese patients. The age at primary diagnosis of esophageal, gastric, pancreatic, and colorectal cancers were all significantly lower among obese patients. The strength of this association was especially pronounced among patients with morbid obesity.

In our linear regression analysis, almost all the common known risk factors for GI cancers including obesity, alcoholism, male gender, and smoking were found to be associated with earlier cancer onset. This is consistent with previous studies that hypothesized a cancer age reduction effect among carcinogenic factors, such as the decreased age of diagnosis of lung cancer found among smokers [19–21]. Among the examined risk factors, obesity, especially morbid obesity, had the most significant impact on early cancer onset for all four cancers. Ulcerative colitis is one of the strongest risk factors for colorectal cancer with the approximate cumulative incidence of 5 to 10 percent after 20 years and 12 to 20 percent after 30 years of disease [22]. In our study, it had the most substantial impact on colon cancer onset age among all known risk factors (8.45 years earlier onset, and odds ratio of 3.08). These findings suggest that the strength of the etiologic factor may be associated with the age at cancer onset. Morbid obesity had a very similar age of onset reduction as ulcerative colitis (7.75 years versus 8.45 years) for colon cancer, suggesting that morbid obesity is a very strong risk factor for early onset of colon cancer. The subgroup of morbidly obese patients is among the fastest growing obese population in the past decades and new data from the CDC show that the prevalence of morbid obesity in US was 7.7% in 2013-2014 [23]. The results of our study highlight the importance and urgency to conduct large prospective studies to definitively establish the relationship between morbid obesity and risk of early onset of cancer. If our results are confirmed by prospective cohort studies, morbidly obese patients may need to have much earlier and more frequent GI cancer screening.

Several limitations exist in using a cross-sectional study design to explore the association between cancer onset age timing and obesity. First, significant undercoding of obesity is likely in our administrative data source. Obesity was only designated as a disease by the American Medical Association (AMA) in 2013, therefore it is likely not regarded as a disease entity worth coding before that time. In addition, a previous study investigating the phenomenon of underreporting obesity in medical records showed that physicians were more likely to document obesity in patient records for those with higher BMI scores or morbidly obese [24]. While CCR has previously been used to conduct obesity epidemiological studies in the literature [25, 26], the undercoding may lead to an exaggeration of the association between obesity and the outcome of interest. Similarly, other clinical risk factors for cancer, such as *Helicobacter pylori* infection, Barrett's disease, etc. were not reliably present in the database, raising risk for residual confounding with the regression models. Another limitation is that the linked PDD-CCR database captured new cancer cases diagnosed in California during the study period only among inpatients, while it was known that some cancer patients were never admitted to the hospital due to advanced cancer stage where inpatient treatment was deemed futile [27]. Another potential limitation of the study is the survival bias, [28], stemming from deaths due to competing causes; that is, subjects with obesity were more likely to die from cardiac disease or stroke at a younger age than control subjects before the development of GI cancers [29]. Studies have shown that obesity, smoking, alcoholism, and diabetes all were associated with a significantly lower life expectancy and might be affected by survival bias [30–32].

## 5. Conclusions

Our study demonstrated that obesity was associated with a three- to five-year reduction and morbid obesity was associated with five to eight-year reduction in the age of diagnosis for four of the most common GI cancers. This finding may be relevant during future efforts in strategizing the optimal age for GI cancer screening for the increasingly prevalent morbidly obese population. Large prospective studies with long-term follow-up are greatly needed to definitively explore the association between cancer onset age and obesity.

## Figures and Tables

**Table 1:** Sample size by cancer type.

Cancer type	Nonobese	Obese	Morbidly obese
Esophageal, no. (%)	8,952 (96.0%)	285 (3.1%)	92 (0.9%)
Pancreatic, no. (%)	26,834 (95.7%)	942 (3.4%)	277 (0.9%)
Colorectal, no. (%)	103,556 (92.4%)	6,325 (5.7%)	2,143 (1.9%)
Gastric, no. (%)	19,416 (95.2%)	760 (3.7%)	225 (1.1%)
Total, no. (%)	158,758 (93.5%)	8,312 (4.9%)	2,737 (1.6%)

**Table 2:** Cancer diagnosis age by cancer type.

Cancer type		Nonobese	Obese	Morbidly obese
Esophageal	Diagnosis age, mean (SD)	68.9 (12.2)	63.7 (11.0^a^)	61.8 (11.2)
	Early onset, no. (%)	552 (6.2%)	28 (9.8%)	15 (16.5%)
Pancreatic	Diagnosis age, mean (SD)	70.6 (12.8)	66.3 (11.7^a^)	62.5 (11.8^b^)
	Early onset, no. (%)	1,632 (6.1%)	79 (8.4%)	32 (11.6%)
Colorectal	Diagnosis age, mean (SD)	69.2 (14.0)	65.3 (11.9^a^)	62.6 (11.6^b^)
	Early onset, no. (%)	9,722 (9.4%)	585 (9.2%)	266 (12.4%)
Gastric	Diagnosis age, mean (SD)	68.5 (14.5)	65.2 (12.4^a^)	63.0 (11.9^b^)
	Early onset, no. (%)	2,197 (11.3%)	88 (11.6%)	36 (16.1%)
All four cancers	Diagnosis age, mean (SD)	69.3 (13.8)	65.4 (11.9^a^)	62.6 (11.6^b^)
	Early onset, no. (%)	24,550 (8.3%)	1,322 (9.7%)	615 (13.9%)

^a^Denotes statistically significant difference between *obese* and *nonobese* diagnosis age (*p* < 0.05). ^b^Denotes statistically significant difference between *morbidly obese* and *obese* diagnosis age (*p* < 0.05).

**Table 3:** Reduction in cancer diagnosis age associated with risk factors (covariates from multivariate linear regression, expressed in covariate ± standard error).

	Esophageal cancer	Pancreatic cancer	Colorectal cancer	Gastric cancer
Morbid obesity^b^	−7.67 ± 1.26^a^	−8.19 ± 1.25^a^	−7.75 ± 0.30^a^	−5.48 ± 0.96^a^
Obesity^b^	−4.73 ± 0.73^a^	−5.35 ± 0.72^a^	−4.56 ± 0.18^a^	−3.25 ± 0.53^a^
Smoking	−1.66 ± 0.26^a^	−1.68 ± 0.26^a^	−0.33 ± 0.11^a^	−0.71 ± 0.25^a^
Alcoholism	−4.34 ± 0.45^a^	−3.70 ± 0.47^a^	−2.90 ± 0.26^a^	−5.29 ± 0.54^a^
Male	−3.24 ± 0.29^a^	−3.32 ± 0.29^a^	−2.52 ± 0.08^a^	−0.94 ± 0.21^a^
Crohn's	—	—	−7.45 ± 0.79^a^	—
Ulcerative colitis	—	—	−8.45 ± 0.56^a^	—
Residual	72.3 ± 0.26	72.1 ± 0.26	70.1 ± 0.06	69.4 ± 0.16

^a^Denotes statistical significance with *p* value < 0.05. ^b^Reduction in cancer onset age represents estimates using nonobese population as reference group.

## Supplementary Materials

Supplementary Materials Supplementary Table 1: demographic and cancer information regarding esophageal cancer patients. Supplementary Table 2: demographic and cancer information regarding gastric cancer patients. Supplementary Table 3: demographic and cancer information regarding pancreatic cancer patients. Supplementary Table 4: demographic and cancer information regarding colorectal cancer patients.
