# Supplementary material for: Obesity Is Associated with Early Onset of Gastrointestinal Cancers in California
Source: J Obes. 2018 Sep 19;2018:7014073. doi: 10.1155/2018/7014073 (PMC6169206; doi:10.1155/2018/7014073)
Supplement: Supplementary Materials — Supplementary Table 1: demographic and cancer information regarding esophageal cancer patients. Supplementary Table 2: demographic and cancer information regarding gastric cancer patients. Supplementary Table 3: demographic and cancer information regarding pancreatic cancer patients. Supplementary Table 4: demographic and cancer information regarding colorectal cancer patients. [file 7014073.f1.pdf]

eTable 1: Demographic and cancer information regarding esophageal cancer patients

	Esophageal Cancer			
	Morbidly Obese n=92 (1.0%)	Obese n = 276 (3.0%)	Non-obese n = 8,961 (96.0%)	P-value*
**Age at Diagnosis, mean ± SD**	61.5 ± 11.0	63.8 ± 11.0	68.9 ± 12.2	<0.01
**Male Gender**	65 (71.4%)	225 (80.9%)	6,575 (74.8%)	0.10
**Race**				
**White**	69 (75.8%)	216 (77.7%)	6,420 (73.0%)	
**Black**	3 (3.3%)	13 (4.7%)	545 (6.2%)	
**Others**	19 (20.9%)	49 (17.6%)	1,827 (20.8%)	0.15
**AJCC Stage at Diagnosis#**				
**0**	48 (1.5%)	2 (1.9%)	48 (1.5%)	0.31
**I**	381 (11.5%)	19 (17.8%)	381 (11.5%)	
**II**	706 (21.4%)	24 (22.4%)	706 (21.4%)	
**III**	772 (23.4%)	24 (22.4%)	772 (23.4%)	
**IV**	1,394 (42.2%)	38 (35.5%)	1,394 (42.2%)	
**Tumor Histology#**				
**Well differentiated**	5 (5.5%)	19 (6.8%)	373 (4.2%)	0.01
**Moderately well differentiated**	25 (27.5%)	106 (38.1%)	2,734 (31.1%)	
**Poorly differentiated**	46 (50.6%)	110 (39.6%)	3,652 (41.5%)	
**Undifferentiated / anaplastic**	1 (1.1%)	3 (1.1%)	143 (1.6%)	
**Smoking**	31 (34.1%)	138 (49.6%)	3,301 (37.6%)	<0.01
**Alcoholism**	4 (4.4%)	25 (9.0%)	771 (8.8%)	0.55

*P-value denotes comparison between obese and non-obese patients.

Missing information regarding staging is present in 5,830 (62.5%); patients; missing information regarding histology is present in 1,978 (21.2%) patients

eTable 2: Demographic and cancer information regarding gastric cancer patients

	Gastric Cancer			
	Morbidly Obese n=224 (1.1%)	Obese n = 760 (3.8%)	Non-obese n = 19,271 (95.1%)	P-value*
**Age at Diagnosis, mean ± SD**	63.1 ± 11.9	65.2 ± 12.4	68.5 ± 14.5	<0.01
**Male Gender**	109 (48.7%)	401 (52.8%)	11,640 (60.4%)	<0.01
**Race**				
**White**	131 (58.5%)	383 (50.4%)	8,593 (44.6%)	<0.01
**Black**	28 (12.5%)	82 (10.8%)	1,380 (7.2%)	
**Others**	65 (29.0%)	295 (38.8%)	9,298 (48.3%)	
**AJCC Stage at Diagnosis#**				
**0**	104 (1.9%)	4 (2.1%)	104 (1.9%)	<0.01
**I**	1,223 (21.9%)	53 (28.2%)	1,223 (21.9%)	
**II**	594 (10.7%)	24 (12.8%)	594 (10.7%)	
**III**	721 (12.9%)	29 (15.4%)	721 (12.9%)	
**IV**	2,932 (52.6%)	78 (41.5%)	2,932 (52.6%)	
**Tumor Histology#**				
**Well differentiated**	9 (4.0%)	48 (6.3%)	772 (4.0%)	<0.01
**Moderately well differentiated**	52 (23.2%)	170 (22.4%)	3,745 (19.4%)	
**Poorly differentiated**	93 (41.5%)	383 (50.4%)	10,268 (53.3%)	
**Undifferentiated / anaplastic**	3 (1.3%)	17 (2.2%)	419 (2.2%)	
**Smoking**	61 (27.2%)	240 (31.6%)	4,236 (22.0%)	<0.01
**Alcoholism**	10 (4.5%)	35 (4.6%)	712 (3.7%)	0.37

*P-value denotes comparison between obese and non-obese patients.
Staging information is missing in 14,425 (71.2%) patients; histology information is missing in 4,183 (20.7%) patients

eTable 3: Demographic and cancer information regarding pancreatic cancer patients

	Pancreatic Cancer			
	Morbidly Obese n=277 (1.0%)	Obese n = 940 (3.4%)	Non-obese n = 26,723 (95.6%)	P-value*
**Age at Diagnosis, mean ± SD**	62.5 ± 11.8	66.3 ± 11.7	70.6 ± 12.8	<0.01
**Early Onset (<50 years)**	32 (11.6%)	79 (8.4%)	1,628 (6.1%)	<0.01
**Male Gender**	101 (32.5%)	394 (41.9%)	13,065 (48.9%)	<0.01
**Race**				
**White**	185 (66.8%)	599 (63.7%)	17,388 (65.1%)	0.05
**Black**	28 (10.1%)	93 (9.9%)	2,064 (7.7%)	
**Others**	64 (23.1%)	248 (26.4%)	7,271 (27.2%)	
**AJCC Stage at Diagnosis#**				
**0**	2 (1.7%)	1 (0.4%)	51 (0.5%)	0.38
**I**	14 (11.8%)	41 (14.5%)	1,106 (10.9%)	
**II**	24 (20.2%)	42 (14.9%)	1,698 (16.7%)	
**III**	6 (5.0%)	29 (10.3%)	1,144 (11.2%)	
**IV**	73 (61.3%)	169 (59.9%)	6,188 (60.7%)	
**Tumor Histology#**				
**Well differentiated**	21 (7.6%)	67 (7.1%)	1,281 (4.8%)	0.01
**Moderately well differentiated**	50 (18.1%)	162 (17.2%)	3,507 (13.1%)	
**Poorly differentiated**	42 (15.2%)	142 (15.1%)	3,467 (13.0%)	
**Undifferentiated / anaplastic**	3 (1.1%)	9 (1.0%)	304 (1.1%)	
**Smoking**	68 (24.6%)	296 (31.5%)	5,644 (21.1%)	<0.01
**Alcoholism**	9 (3.3%)	46 (4.9%)	1,131 (4.2%)	0.48

	Pancreatic Cancer			

*P-value denotes comparison between obese and non-obese patients.
Missing information regarding staging is present in 17,462 (62.5%) patients; missing information regarding histology is present in 5,979 (21.4%) patients

eTable 4: Demographic and cancer information regarding colorectal cancer patients

	Colorectal Cancer			
	Morbidly Obese n=2,109 (1.92%)	Obese n=6,232 (5.66%)	Non-obese n=101,718 (92.42%)	P-value*
**Age at Diagnosis, mean ± SD**	62.6 ± 11.6	65.3 ± 11.9	69.2 ± 14.0	<0.01
**Early onset (<50 y/o)**	262 (12.4%)	576 (9.2%)	9,604 (9.4%)	<0.01
**Male Gender**	890 (42.2%)	2,959 (47.5%)	51,347 (50.5%)	<0.01
**Race**				
**White**	1,428 (67.7%)	4,073 (65.4%)	66,569 (65.4%)	<0.01
**Black**	210 (10.0%)	595 (9.6%)	7,119 (7.0%)	
**Others**	471 (22.3%)	1,564 (25.1%)	28,030 (27.6%)	
**AJCC Stage at Diagnosis#**				
**0**	39 (8.2%)	81 (6.6%)	1,536 (6.3%)	<0.01
**I**	141 (29.6%)	377 (30.6%)	6,266 (25.9%)	
**II**	114 (24.0%)	318 (25.8%)	5,901 (24.4%)	
**III**	86 (18.1%)	252 (20.4%)	4,234 (17.5%)	
**IV**	96 (20.2%)	205 (16.6%)	6,302 (26.0%)	
**Tumor Histology#**				
**Well differentiated**	199 (9.4%)	578 (9.3%)	8,604 (8.5%)	<0.01
**Moderately well differentiated**	1,283 (60.8%)	3,841 (61.6%)	59,637 (58.6%)	
**Poorly differentiated**	335 (15.9%)	988 (15.9%)	17,260 (17.0%)	
**Undifferentiated / anaplastic**	23 (1.1%)	68 (1.1%)	1,231 (1.2%)	
**Smoking**	448 (21.2%)	1,676 (26.9%)	18,734 (18.4%)	<0.01
**Alcoholism**	49 (2.3%)	188 (3.0%)	2,606 (2.6%)	0.12
**Ulcerative Colitis**	18 (0.9%)	31 (0.5%)	548 (0.5%)	0.56

*P-value denotes comparison between obese and non-obese patients.
Missing information regarding staging is present in 68,786 (62.5%); patients; missing information regarding histology is present in 23,332 (21.2%) patients
